# Sub-Graph Regularization on Kernel Regression for Robust Semi-Supervised Dimensionality Reduction

**DOI:** 10.3390/e21111125

**Published:** 2019-11-15

**Authors:** Jiao Liu, Mingbo Zhao, Weijian Kong

**Affiliations:** 1 School of Management Studies, Shanghai University of Engineering Science, Shanghai 201600, China; liujiaolndx@163.com; 2 School of Information Science and Technology, Donghua University, Shanghai 201620, China

**Keywords:** kernel regression, semi-supervised learning, dimensionality reduction, anchor graph regularization

## Abstract

Dimensionality reduction has always been a major problem for handling huge dimensionality datasets. Due to the utilization of labeled data, supervised dimensionality reduction methods such as Linear Discriminant Analysis tend achieve better classification performance compared with unsupervised methods. However, supervised methods need sufficient labeled data in order to achieve satisfying results. Therefore, semi-supervised learning (SSL) methods can be a practical selection rather than utilizing labeled data. In this paper, we develop a novel SSL method by extending anchor graph regularization (AGR) for dimensionality reduction. In detail, the AGR is an accelerating semi-supervised learning method to propagate the class labels to unlabeled data. However, it cannot handle new incoming samples. We thereby improve AGR by adding kernel regression on the basic objective function of AGR. Therefore, the proposed method can not only estimate the class labels of unlabeled data but also achieve dimensionality reduction. Extensive simulations on several benchmark datasets are conducted, and the simulation results verify the effectiveness for the proposed work.

## 1. Introduction

Dimensionality reduction is an important issue when handing high-dimensional data in many real-world applications, such as image classification, text recognition, etc. In general, dimensionality reduction is achieved by finding a linear or nonlinear projection matrix that casts the original high-dimensional data into a low-dimensional subspace so that the computational complexity can be reduced and the key intrinsic information can be preserved [1,2,3,4,5,6,7,8,9,10]. Principal component analysis (PCA) and linear discriminant analysis (LDA) [11] are two of the most widely-used methods for dimensionality reduction. PCA is achieved by finding a projection matrix along the maximum variance of the dataset with the best reconstruction. While LDA is utilized to search for the optimal direction ensuring that the dataset in the reduced subspace can maximize the between-class scatter while minimizing the within-class scatter. As LDA is a supervised approach, it generally outperforms PCA by giving sufficient labeled information.

A key problem is that obtaining a large amount of labeled data is time-consuming and expensive. On the other hand, unlabeled data may be abundant in some real world applications. Therefore, semi-supervised learning (SSL) approaches have become increasingly important in the area of pattern recognition and machine learning [1,2,4,12,13,14]. Over the past decades, according to the manifold or clustering assumptions—i.e., nearby data likely have the same labels [1,2,4]—graph based SSL is one of the most popular methods in the aspect of SSL, which includes the manifold regularization (MR) [3], learning with local and global consistency (LGC) [2] and Gaussian fields and harmonic functions (GFHF) [1] methods. All of these utilize labeled and unlabeled sets to formulate a graph for approximating the geometry of data manifolds [5].

The above graph-based SSL can be usually divided into two categorizations: The first is the inductive learning method and the second is the transductive learning one. The transductive learning methods aim to propagate the labeled information via a graph [1,2,4], so that the labels of an unlabeled set are estimated. However, a key problem for transductive learning methods is that they cannot estimate the class labels of new incoming data, therefore suffering from the out-of-sample problem. In constrast, the inductive learning methods, known as MR [3] and Semi-supervised Discriminant Analysis (SDA) [5], aim to study a decision function for classification on the original data space, so that they can reduce the dimensionality as well as naturally solve out-of-sample problems.

It can be noted that the graph in SSL tends to be a *k* nearest neighborhood (*k*NN) based graph that is first to find the *k*-neighborhoods of each data [15,16,17] and then define a weight matrix measuring the similarity between any pair-wise data [1,2,4,18,19,20,21]. However, *k*NN graph has a key limit in that it cannot be scalable to a large-scale dataset, as the computational complexity for searching the *k* neighborhoods of data is $O\left(kn^{2}\right)$, which is not linear with *n*. To solve this problem, Liu et al. [22,23] proposed an efficient anchor graph (AGR), where each data point is first to find the *k* neighborhoods of anchor points, then the graph is constructed by the inner product of coefficients between the data and anchors, through which the class labels can be inferred from anchors to the whole dataset. As a result, the computational complexity can be greatly reduced. While there are different ways to build the adjacency matrix *S* in AGR [24,25,26], we argue that most of them are developed intuitively and lack a probability explanation. In addition, AGR cannot directly infer the class labels of incoming data.

In this paper, we aim to enhance AGR by solving the above problems. From the element concept idea of AGR, we point that the anchors should have the same probability distribution to those of data points, as the anchors refer to the data that can roughly approximate the distribution of data points. Based on this assumption, we then analyze *S* from the stochastic view and further extend it to be doubly-stochastic. As a result, the distribution of anchors is the same to those of data points, and the updated *S* can be treated as a transition matrix, where each value in *S* can be viewed as a transition probability value between any data point and anchor point. Benefiting from *S*, we then develop a sub-graph regularized framework for SSL. The new sub-graph is constructed by *S* in an efficient way and can preserve the geometry of data structure. Accordingly, an SSL strategy based on such a sub-graph is also developed, which is first to infer the labels of anchors and then to calculate those of the training data. The is quite different from conventional graph-based SSL, which is directly to infer the class labels of datasets on the whole graph and may result in a huge computational cost if the dataset is large-scale. However, this SSL strategy is efficient and suitable for handling a large-scale dataset. The experiments on extensive benchmark datasets show the effectiveness and efficiency of the proposed SSL method.

The main contributions of this paper are given as follows:

(1) We develop a doubly-stochastic *S* that measures the similarity between data points and anchors. The new updated *S* has probability means and can be viewed as transition probability between data points and anchors. In addition, the proposed *S* is also a stochastic extension to the ones in AGR.
(2) We develop a sub-graph regularized framework for SSL. The new sub-graph is constructed by *S* in an efficient way and can preserve the geometry of the data manifold.
(3) We also adopt a linear predictor for inferring the class labels of new incoming data, which can handle out-of-sample problems. In addition, the computational complexity of this linear predictor is linear with the number of anchors, and hence is efficient.

The organization of the paper is as follows: In Section 2, basic notations and reviews for SSL are provided; in Section 3, the proposed model for graph construction and SSL are developed. In Section 4, we conduct extensive simulations, and give our final conclusions in Section 5.

## 2. Notations and Preliminary Work

### 2.1. Notations

Let $X=\left[X_{l},X_{u}\right]\in R^{d\times \left(l+u\right)}$ be the data matrix, where *d* presents the feature number, *l* and *u* are the number of labeled and unlabeled sets, respectively, so that $X_{l}$ and $X_{u}$ are respectively the labeled and unlabeled sets, $Y=\left[y_{1},y_{2},\ldots ,y_{l+u}\right]\in R^{c\times \left(l+u\right)}$ be the one hot labels of data, $F=\left[f_{1},f_{2},\ldots ,f_{l+u}\right]\in R^{c\times \left(l+u\right)}$ is the predicted label matrix satisfying $0\leq f_{ij}\leq 1$.

### 2.2. Review of Graph Based Semi-Supervised Learning

We will review the prior graph based SSL methods. Two well-known methods for SSL include LGC [1] and GFHF [2]. The objective of LGC and GFHF can be given as:

(1) $$\begin{array}{c} g_{L}\left(F\right)=\frac{1}{2}\sum_{i,j=1}^{l+u}{∥\frac{f_{i}}{\sqrt{D_{ii}}}-\frac{f_{j}}{\sqrt{D_{jj}}}∥}_{F}^{2}W_{ij}+\lambda \sum_{i=1}^{l+u}{∥f_{i}-y_{i}∥}_{F}^{2}\\ g_{G}\left(F\right)=\frac{1}{2}\sum_{i,j=1}^{l+u}{∥f_{i}-f_{j}∥}_{F}^{2}W_{ij}+\lambda_{\infty }\sum_{i=1}^{l}{∥f_{i}-y_{i}∥}_{F}^{2} \end{array}$$

where λ is a balancing parameter that controls the trade off between the label fitness and the manifold smoothness. $\lambda_{\infty }$ is a large value such that $\sum_{i=1}^{l}\left|\right|f_{i}-y_{i}{\left|\right|}_{F}^{2}=0$, or $f_{i}=y_{i}$, ∀i=1,2,…,l.

### 2.3. Anchor Graph Regularization

Anchor graph regularization (AGR) is an efficient graph based learning method for large-scale SSL. In detail, let $A=\left\{a_{1},a_{2},\ldots a_{m}\right\}\in R^{d\times m}$ be the anchor point set, $G=\left\{g_{1},g_{2},\ldots g_{m}\right\}\in R^{c\times m}$ be the label matrix of *A*, $Z\in R^{m\times n}$ be the weight matrix measuring the similarity between each $x_{j}$ and $a_{i}$ with constraints $Z_{ij}\geq 0$ and $\sum_{i=1}^{m}Z_{ij}=1$, which is usually formulated by the kernel weights or the local reconstructed strategy making the computational complexity for both two strategies linear with the data number. Then, the label matrix *F* can be estimated as:

(2) $$f_{j}=\sum_{i=1}^{m}g_{i}Z_{ij},$$

so that AGR is to minimize the following objective function:

(3) $$\begin{array}{c} J\left(G\right)=\sum_{j=1}^{l}{∥Gz_{j}-y_{j}∥}_{F}^{2}+\frac{\gamma }{2}\sum_{i,j=1}^{n}W_{ij}^{a}{∥Gz_{i}-Gz_{j}∥}_{F}^{2}\\ \;\;\;\;={∥GZ_{l}-Y_{l}∥}_{F}^{2}+\gamma Tr\left(GZ\left(I-W^{a}\right)Z^{T}G^{T}\right)\\ \;\;\;\;={∥GZ_{l}-Y_{l}∥}_{F}^{2}+\gamma Tr\left(GL^{r}G^{T}\right) \end{array},$$

where the first term is the loss function and the second term is the manifold regularized term, $W^{a}=Z^{T}\Delta^{-1}Z\in R^{n\times n}$ is the anchor graph, and $\Delta \in R^{m\times m}$ is a diagonal matrix with each element satisfying $\Delta_{ii}=\sum_{j=1}^{n}Z_{ij}$. It can be easily proven that $W^{a}$ is doubly-stochastic, hence it has probability meaning. In addition, given two data points $x_{i}$ and $x_{j}$ with common anchor points, it follows $W_{ij}^{a}>0$; otherwise $W_{ij}^{a}=0$. This indicates that the data points with common anchor points have similar semantic concepts hence $W^{a}$ can characterize the semantic structure of datasets. $L^{r}=Z\left(I-W^{a}\right)Z^{T}\in R^{m\times m}$ is the reduced Laplacian matrix, $Z_{l}\in R^{m\times l}$ is formed by the first *l* columns of *Z*. Here, we can see that although AGR is performed with a regularization term on all data points, it is equivalent to being regularized on anchor points with a reduced Laplacian matrix $L^{r}$. Finally, the labels of data points can be inferred from those of anchor points, where the computational complexity can be reduced to $O\left(n\right)$. Therefore, both graph construction and the regularized procedure in AGR are efficient and scalable to a large-scale dataset.

## 3. A Sub-Graph Regularized Framework for Efficient Semi-Supervised Learning

### 3.1. Analysis of Anchor Graph Construction

The key point for anchor graph construction is to define the weight matrix for measuring the similarity between each data point and anchor data. A typical way is to use kernel regression [22]:

(4) $$S_{ij}=\frac{K_{\delta }\left(x_{i},b_{j}\right)}{\sum_{s\in \langle i\rangle }K_{\delta }\left(x_{i},b_{s}\right)}\forall s\in \langle i\rangle$$

where δ is the bandwidth of Gaussian function and $\langle i\rangle$ denotes the indices of the *k* neighborhood anchors of $x_{i}$. Obviously, we have $S^{T}1_{q}=1_{n}$, where $1_{n}\in R^{n\times 1}$ and $1_{q}\in R^{q\times 1}$ is the column vectors with *n* and *q* ones, respectively, so that the sum of each column of *S* is equal to 1. This means $S_{ij}$ can be viewed as a probability value $P\left(b_{i}|x_{j}\right)$, which represents the transferred probability from $x_{j}$ to $b_{j}$. Then, following the Bayes rule, we have:

(5) $$P\left(b_{i}\right)=\sum_{j=1}^{n}P\left(x_{j}\right)P\left(b_{i}|x_{j}\right)\approx \frac{1}{n}P\left(b_{i}|x_{j}\right)$$

where $P\left(x_{j}\right)\approx 1/n$ follows a uniform distribution based on the strong law of large number n→∞. In addition, since the anchors are also sampled from the dataset, we can further assume $P\left(b_{i}\right)$ also follows a uniform distribution, i.e., $P\left(b_{i}\right)=1/q$. With these assumptions, we have:

(6) $$\begin{array}{c} \left\{\begin{array}{cc} P\left(b_{i}\right) & =1/q,P\left(x_{j}\right)=1/n\\ P\left(b_{i}\right) & =\sum_{j=1}^{n}P\left(x_{j}\right)P\left(b_{i}|x_{j}\right) \end{array}\right\}\\ \Rightarrow \sum_{j=1}^{n}P\left(b_{i}|x_{j}\right)=\frac{n}{q}\Rightarrow S_{i.}1_{n}=\sigma \end{array}$$

where $S_{i}$ is the *i*-th row of *S* and σ=n/q is a fixed value so that $S1_{n}=\left(n/q\right)1_{q}=\sigma 1_{q}$. We thereby have two constraints on *S*, i.e., $S^{T}1_{q}=1_{n}$ and $S1_{n}=\sigma 1_{q}$ (the advantages will be shown in the next subsection). Our goal is to calculate a weight matrix *S* that follows the above constraints so that *S* has clear stochastic meaning.

Fortunately, this can be simply achieved by iteratively normalizing *S* both in row and column, i.e.,

(7) $$S^{0}\overset{P_{r}\left(\right)}{\to }S^{1}\overset{P_{c}\left(\right)}{\to }S^{1}\overset{P_{r}\left(\right)}{\to }S^{2}\overset{P_{c}\left(\right)}{\to }S^{2}\to \cdots$$

where $P_{c}\left(S\right)=S\Delta_{c}^{-1}$ and $P_{r}\left(S\right)=\Delta_{r}^{-1}S$, $\Delta_{c}=diag\left(1S\right)\in R^{\left(l+u\right)\times \left(l+u\right)}$ and $\Delta_{r}=diag\left(S1\right)\in R^{q\times q}$. Acutally, the above iterative procedure is equivalent to solving the following optimization problem:

(8) $$\min_{S}{∥S-S_{0}∥}_{F}^{2}\;s.t.\;S\geq 0,\;S^{T}1_{q}=1_{n},\;S1_{n}=\sigma 1_{q}$$

where $S_{0}$ is the initial *S* as calculated in Equation (4). Equation (8) involves an instance of quadratic programming (QP), which can be divided into two convex sub-problems:

(9) $$\min_{S}{∥S-S_{0}∥}_{F}^{2}\;s.t.\;S\geq 0,\;S^{T}1_{q}=1_{n}$$

(10) $$\min_{S}{∥S-S_{0}∥}_{F}^{2}\;s.t.\;S\geq 0,\;S1_{n}=\sigma 1_{q}.$$

By the above derivations, the initial QP problem in Equation (8) is tackled by successively alternating between two sub-problems in Equations (9) and (10). This alternate optimization procedure will converge due to Von-Neumann’s lemma [27,28]. In addition, Von-Neumann’s lemma guarantees that alternately solving the sub-problems in Equations (9) and (10) with the current solution is theoretically guaranteed to converge to the global optima of Equation (8).

### 3.2. Sub-Graph Construction

We have now obtained *q* anchors and the coefficient $s_{j}$ of each data $x_{j}$. The weight matrix *S* reflects the affinities between data points and anchors, i.e., X≈BS. If we further assume such affinities in the original high-dimensional dataset can be preserved in the low-dimensional class labels, then we have F≈ZS, where $Z=\left[z_{1},z_{2},\ldots ,z_{q}\right]\in R^{c\times q}$ represents the class labels of anchors *B*. This indicates that the class labels of the dataset can be easily obtained by F=ZS, given that the class labels of anchors have already been inferred. Since the number of anchors is smaller than that of the dataset, the computational cost for calculating *Z* can be much lower than directly calculating *F* in certain conventional graph-based SSL methods. We thereby present an efficient method for semi-supervised learning, in which we aim to develop a sub-graph regularized (SGR) framework for semi-supervised learning by utilizing the information of anchors.

Here, in order to develop our proposed sub-graph SSL method, we need to first construct a sub-graph on the set of anchors and define the adjacency matrix to measure the similarity between any two anchors. There are many approaches to construct the graph by utilizing the anchors, such as conventional *k*NN graph [1,18,20,21]. However, intuitively, we will design the adjacency matrix $W^{d}\in R^{q\times q}$ by using *S* as follows:

(11) $$W^{d}=\frac{1}{\sigma }SS^{T}.$$

It can be easily proven that $W^{d}1_{q}=\left(1/\sigma \right)SS^{T}1_{q}=\left(1/\sigma \right)S1_{n}=1_{q}$. This indicates $W_{d}$ is a doubly-stochastic matrix. Therefore, the above graph construction can be theoretically derived by a probabilistic means. More straightforward, it can be easily noted that $W^{d}$ in Equation (11) is an inner product of *S* with each element $W_{ij}^{d}=s_{i}^{r}$, where $s_{i}^{r}{s_{j}^{r}}^{T}$ and $s_{j}^{r}$ are the *i*-th and *j*-th rows of $S=\{s_{1}^{r},s_{2}^{r},\ldots ,s_{q}^{r}\}$. This indicates that the rows of *S* are denoted as the representations of anchors. In addition, given $b_{i}$ and $b_{j}$ share more common data points choosing them as anchors, their corresponding $s_{i}^{r}$ and $s_{j}^{r}$ will be similar and $W_{ij}^{d}$ will become a large value; To the constrast, $W_{ij}^{d}$ will be equal to 0, if $b_{i}$ and $b_{j}$ do not share any data points. Therefore $W^{d}$ derived in Equation (11) can be viewed as an adjacency matrix to measure the similarity between any two anchors.

### 3.3. Efficient Semi-Supervised Learning via Sub-Graph Construction

With the above graph construction, we then develop our sub-graph model for efficient semi-supervised learning. Since the number of anchors is much smaller than that of the dataset, our goal is first to estimate the labels of anchors *Z* from labeled data via the sub-graph model, and then to calculate those of unlabeled samples by the weight matrix. Here, we first give the objective function of the proposed sub-graph regularized framework for calculating the class labels of anchors as follows:

The first term in Equation (12) is to measure the smoothness of estimated labels on the graph, while the second term is to measure how the estimated labels are consistent original labels, and the third one is a Tikhonov regularization term to avoid the singularity of possible solutions. $\eta_{A}$ and $\eta_{I}$ are the parameters balancing the tradeoff of the three terms. By conducting the derivation of $J\left(Z\right)$ with regard to *Z*, we can calculate the class labels for anchors as follows:

(12) $$Z^{*}=YUS^{T}{\left(SUS^{T}+\eta_{A}I+\eta_{I}L^{d}\right)}^{-1}$$

where *U* is a diagonal matrix where the first *l* and the remaining *u* element are 1 and 0, respectively, $L^{d}$ is the graph Laplacian matrix of $W^{d}$. Following Equation (13), we can observe that key computations for $Z^{*}$ are the inverse of $S_{l}S_{l}^{T}+\eta_{I}L^{d}+\eta_{A}I$, where the complexity is $O\left(q^{3}\right)$. Note that q≪l+u, calculating *Z* can be much smaller than directly calculating *F* as in LGC and GFHF. Finally, the class labels of the dataset can be calculated by

(13) $$F=Z^{*}S=YUS^{T}{\left(SUS^{T}+\eta_{I}L^{d}+\eta_{A}I\right)}^{-1}S.$$

The basic steps of the proposed SGR are in Algorithm 1.

**Algorithm 1:** The proposed SGR.
**1** **Input**: Data $X\in R^{D\times \left(l+u\right)}$, label matrix $Y\in R^{c\times \left(l+u\right)}$, the number of anchors *q* and other parameters.**2** From *S* as Equation (8).**3** Form sub-graph weight matrix as $SS^{T}$ in Equation (11).**4** Estimate the label matrix of anchors $Z^{*}=YUS^{T}{\left(SUS^{T}+\eta_{I}L^{d}+\eta_{A}I\right)}^{-1}$ as in Equation (12).**5** Estimate the label matrix of dataset by $F=Z^{*}S$.**6** **Output**: The predicted label matrix of anchors and dataset $Z\in R^{c\times q}$, $F\in R^{c\times \left(l+u\right)}$, respectively.


### 3.4. Out-of-Sample Extension via Kernel Regression

The proposed SGR can be used to estimate the labels of unlabeled data. It cannot directly infer the labels of new data. One way to handle such problems is to find a linear projective model by regressioning anchors *B* on *Z*, i.e.,:

(14) $$V=\arg\min_{V,b}{∥V^{T}B+b^{T}e-Z∥}_{F}^{2}+\gamma {∥Z∥}_{F}^{2}$$

where $V\in R^{d\times c}$ is the projection and *b* is the bias term. Though this linearization assumption $Z=V^{T}B+b^{T}e$ provides an effective and efficient solution to the out-of-sample problem. However it is not able to fit the nonlinear distribution. Therefore, we solve the above problem in two ways: (1) We combine the objective function of SGR and the regression term to form a unified framework, so that the class labels of *Z*, the projection *V*, and the bias *b* can be simultaneously calculated; (2) we utilize the kernel trick to search a nonlinear projection. Specifically, we give the objective function as:

(15) $$\begin{array}{c} J\left(V,Z,b\right)=\min_{V,Z,b}\sum_{j=1}^{l}{∥Zs_{j}-y_{j}∥}_{F}^{2}+\eta_{A}{∥V∥}_{F}^{2}\\ \;\;\;\;\;\;\;\;\;+\eta_{R}{∥V^{T}\varphi \left(X\right)+b^{T}e-Z∥}_{F}^{2}\\ \;\;\;\;\;\;\;\;\;+\eta_{I}\sum_{i,j=1}^{q}W_{ij}^{d}{∥z_{i}-z_{j}∥}_{F}^{2}. \end{array}$$

It should be noted that $\varphi \left(B\right)$ is only implicit and not available. To calculate the optimal *V*, we have to involve some restrictions. In detail, let *V* have a linear combination of $\varphi \left(B\right)$, i.e., $V=\varphi \left(B\right)A$, where $A\in R^{q\times c}$ is the coefficient for *V*, then:

(16) $$\begin{array}{c} J\left(V,Z,b\right)=\min_{V,Z,b}\sum_{j=1}^{l}{∥Zs_{j}-y_{j}∥}_{F}^{2}+\eta_{A}Tr\left(A^{T}KA\right)\\ \;\;\;\;\;\;\;\;\;+\eta_{R}{∥A^{T}K+b^{T}e-Z∥}_{F}^{2}\\ \;\;\;\;\;\;\;\;\;+\eta_{I}\sum_{i,j=1}^{q}W_{ij}^{d}{∥z_{i}-z_{j}∥}_{F}^{2} \end{array}$$

where *K* represents the kernel matrix and we can select Gaussian kernel. By setting the derivatives of Equation (16), if follows:

(17) b=1qZT−1qKA1qZT−1qKA1q1qT1q1qTA=KLcKT+ηK−1KLcZTZ=YUSTSUST+ηILd+ηRLr−1

where $\eta =\eta_{I}/\eta_{R}$, $L_{c}=I-{1_{q}}^{T}1_{q}/1_{q}{1_{q}}^{T}\;$ is to subtract the mean of all data, $L^{r}=L_{c}-L_{c}K^{T}{\left(KL_{c}K^{T}+\eta I\right)}^{-1}KL_{c}$. Here, denote *x* as a new coming data and $x_{k}$ as its kernel representation, its projected data *t* can be given $t=V^{T}x_{k}+b$ and the label of *x* is estimated as:

(18) $$c_{t}=\arg\max_{i}t\left(i\right)$$

**Figure 1:**
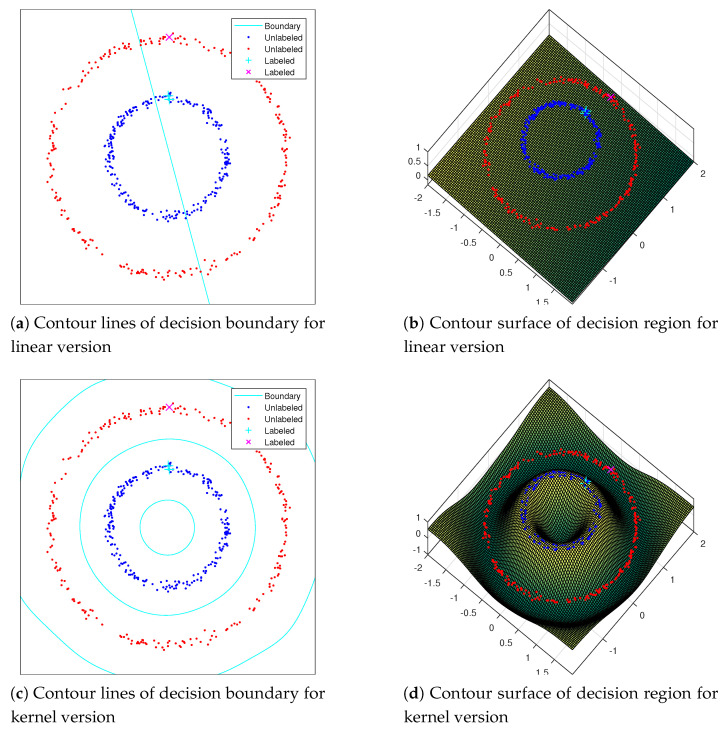
Out-of-sample extension: two-cycle dataset in $\left\{\left(x,y\right)|x\in \left[-2,2\right],y\in \left[-2,2\right]\right\}$. (**a**,**c**) the contour lines of the decision boundary; (**b**,**d**) the contour surface is the estimated label values in the region. In this experiment, the figures in the upper row represent the results by using a linear prediction model $Z=V^{T}B+b^{T}e$, while those on the bottom row represent the results by using a kernel based prediction model $Z=V^{T}\varphi \left(B\right)+b^{T}e$. Clearly, the kernel prediction model is much better than the linear prediction model since the two-cycle dataset follows a nonlinear distribution.

One toy model example for verifying out-of-sample extensions can be given in Figure 1. In this toy example, we annotate two datasets as labeled sets in each class. We then infer the labels in the region $\left\{\left(x,y\right)|x\in \left[-2,2\right],y\in \left[-2,2\right]\right\}$ by out-of-sample extension both in the linear version and kernel version. The experiment results show that the decision boundary learned by the kernel version is satisfied, since they are both consistent with the data manifold. While the linear version fails to handle the task, due to the two-cycle dataset following a nonlinear distribution.

Note that the proposed method includes three stages of training: (1) initialize the anchors by *k*-means; (2) construct the sub-graph $w^{d}$; (3) perform SSL. Here, the computational cost of k-means in the first stage is $O\left(q\left(l+u\right)\right)$, while the one for sub-graph construction and SSL strategy in the second and third stage are $W^{d}$ is $O\left(q\left(l+u\right)\right)$ and $O\left(q^{3}+\left(l+u\right)q\right)$, respectively. The summary of the computational complexity is in Table 1, from which we can see that if we use a fixed *q* (q≪l+u) anchors for large scale dataset, the computational complexity of proposed SGR scales linearly with l+u, which indicates the proposed SGR is suitable for handling large-scale data.

It should be noted a recent work, [29], has proposed another SSL method based coupled graph Laplacian regularization, which is similar to our proposed work. The main advantages for our proposed work compared to [29] can be issued as follows: (1) The proposed constructed graph is doubly-stochastic, so that the constructed graph Laplacian is normalized in each row or column. For the coupled graph Laplacian rigorization, their constructed graph may not be doubly-stochastic; (2) the proposed work can directly handle out-of-sample problems by projecting the newly-coming data on the projection matrix so that the class membership of newly-coming data can be inferred. While for the coupled graph Laplacian regularization, it does not consider this point.

## 4. Experiments

### 4.1. Toy Examples for Synthetic Datasets

**Figure 2:**
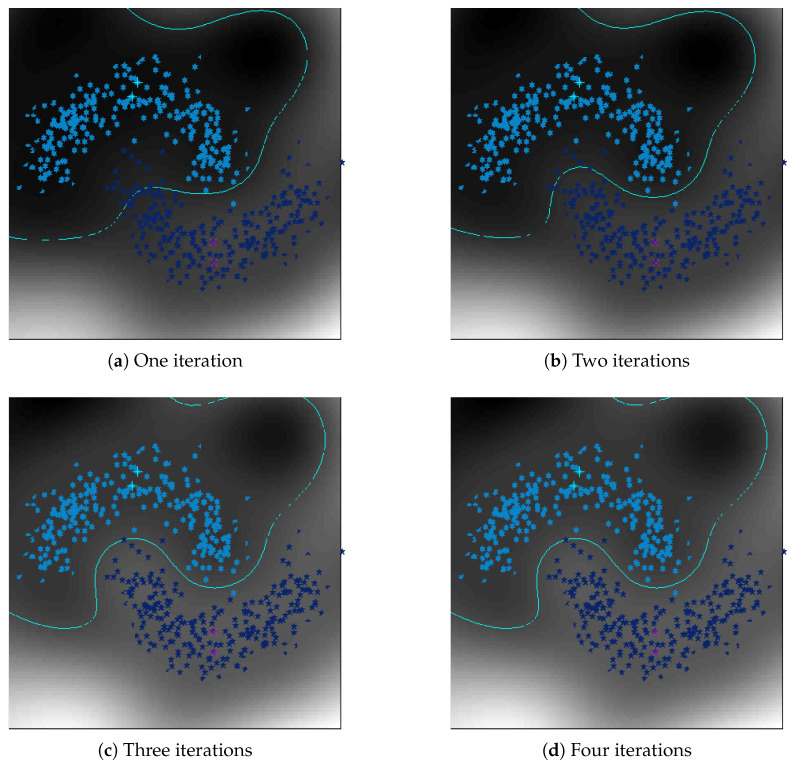
Gray image of reduced space learned by the proposed method: two-moon dataset.

We will first show the iterative approach of the proposed method can adaptively reduce the bias of a data manifold, where a dataset of two classes with noises is generated with a half-moon distribution in each class. Here, we use a kernel version of the proposed method to learn the classification model to handle such nonlinear distribution. Figure 2 shows the decision surfaces and boundaries obtained by the proposed method during the iterations. From Figure 2, we can observe that for the two-moon dataset, the results converge fast by only using four iterations. In Figure 2, we can observe that by initially treating each local regression term equal, the boundary learned by the proposed method cannot well separate the two classes as there are many mis-classified data points. However, during the iterative rewrighted process, the converged boundary in Figure 2 after four iterations can be more and more accurate and distinctive due to the reason that the biases caused by the noisy data are seriously reduced.

### 4.2. Description of Dataset

**Figure 3:**
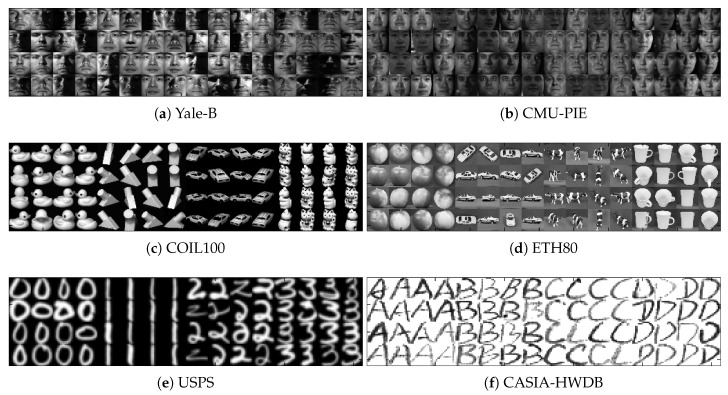
Sample images of real-world datasets: Yale-B, Carnegie Mellon University Pose, Illumination and Expression (CMU-PIE), Columbia Object Image Library 100 (COIL-100), Eidgenössische Technische Hochschule 80 (ETH80), U. S. Post Station (USPS) digit image and Chinese Academy of Sciences, Institute of Automation, Hand-Written Digit Base (CASIA-HWDB) datasets.

In this section, we will utilize six real-world datasets for verification. The six datasets are the Extended Yale-B, Carnegie Mellon University Pose, Illumination and Expression (CMU-PIE), Columbia Object Image Library 100 (COIL-100), Eidgenössische Technische Hochschule 80 (ETH80), U. S. Post Station (USPS) digit image and Chinese Academy of Sciences, Institute of Automation, Hand-Written Digit Base (CASIA-HWDB) datasets. For each dataset, we only select 5%, 10%, 15%, and 20% of the data points to formulate a labeled set randomly, 20% of the data to formulate a test set, and the remaining ones to formulate an unlabeled set. The information of the data and sampled images can be observed in Table 2 and Figure 3, respectively.

### 4.3. Image Classification

We will show the effectiveness of the proposed SGR for image classification. The experiment settings are as follows [36,37]: For most SSL methods, e.g., LGC, Special Label Propagation (SLP), Linear Neighborhood Propagation (LNP), AGR, Efficient Anchor Graph Regularization (EAGR) and MR, the parameter *k* for constructing the *k*NN graph is determined by five-fold cross validation, which is chosen from 6 to 20. For LGC, LNP AGR, and EAGR, the regularized parameter is needed to set, which is determined from $\left\{10^{-6},10^{-3},10^{-1},1,10,10^{3},10^{6}\right\}$. The average accuracies of over 50 random splits with changed numbers of labeled data are shown in Table 3, Table 4, Table 5, Table 6, Table 7 and Table 8. From the classification results, we have:

(1) For almost all methods, the classification results increase given that the number of labeled data increases. For instance, the results of SGR will increase 15% as the number of labeled data is increased from 5% to 20% in most cases. This can almost get 17% increase in CASIA-HWDB dataset. In addition, the classification results will not increase given the number of labeled samples are sufficient especially in the cases of COIL100, USPS, and ETH80 datasets;

(2) The proposed SGR can outperform other methods in all cases. For instance, SGR can achieve 5%–9% superiority over SLP, LNP, and MR in almost all cases. Especially in the CASIA-HWDB dataset, this improvement can even achieve 9%. AGR and EAGR can obtain competitive results as SGR by tuning the parameters. However, the proposed SGR can automatically adjust them while achieving satisfying results;

(3) The accuracies of the unlabeled set outperform those of the test set. This is because the testing data are not utilized for training. However, the accuracies of the test set are still good showing that SGR is able to handling the new incoming data.

### 4.4. Parameter Analysis with Different Numbers of Anchors

**Figure 4:**
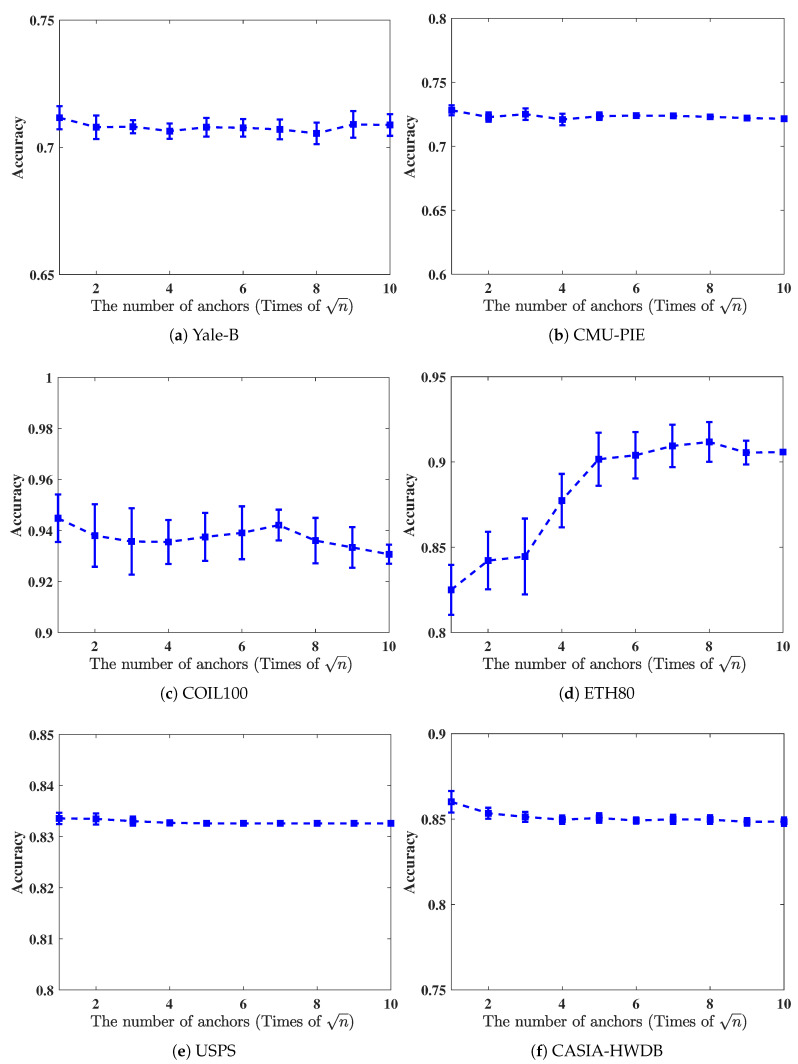
Classification accuracies over different numbers of anchors.

In this subsection, we will verify the accuracies of SGR against different numbers of anchors. In this study, we selected 5% data to formulate a labeled set and the remaining ones to formulate an unlabeled set. Then, in Figure 4, we give the accuracy curve of SGR under different numbers of anchors, where the candidate set is chosen from $\sqrt{n}$ to $10\sqrt{n}$.

From Figure 4, we can see that in ETH80 dataset, the classification results increase when the number of anchors increase. However, the accuracies will not increase anymore given sufficient number of anchors, such as $10\sqrt{n}$. Here, $10\sqrt{n}$ is still much smaller compared with that of original data. For other datasets, the classification accuracies have no change and are less sensitive to the number of anchors.

### 4.5. Image Visualization

**Figure 5:**
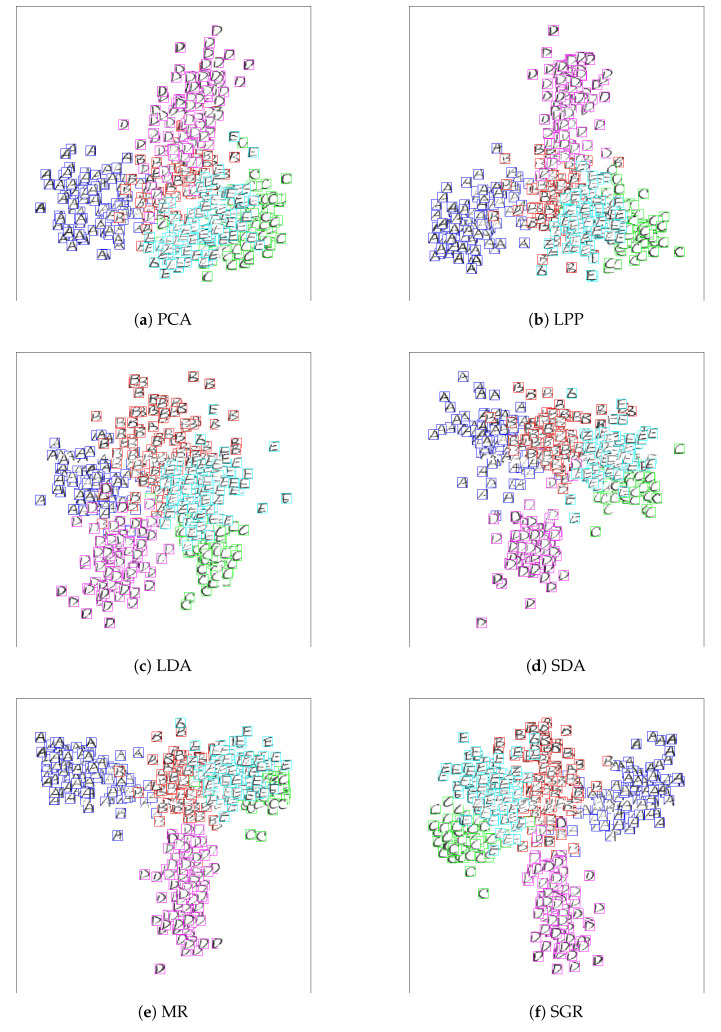
Visualization performance of different methods: Five letters images from CASIA-HWDB: Principal Component Analysis (PCA), Locality Preserving Projection (LPP), Linear Discriminant Analysis (LDA), Semi-supervised Discriminant Analysis (SDA), Manifold Regularization (MR) and Sub-Graph Regularization (SGR).

**Figure 6:**
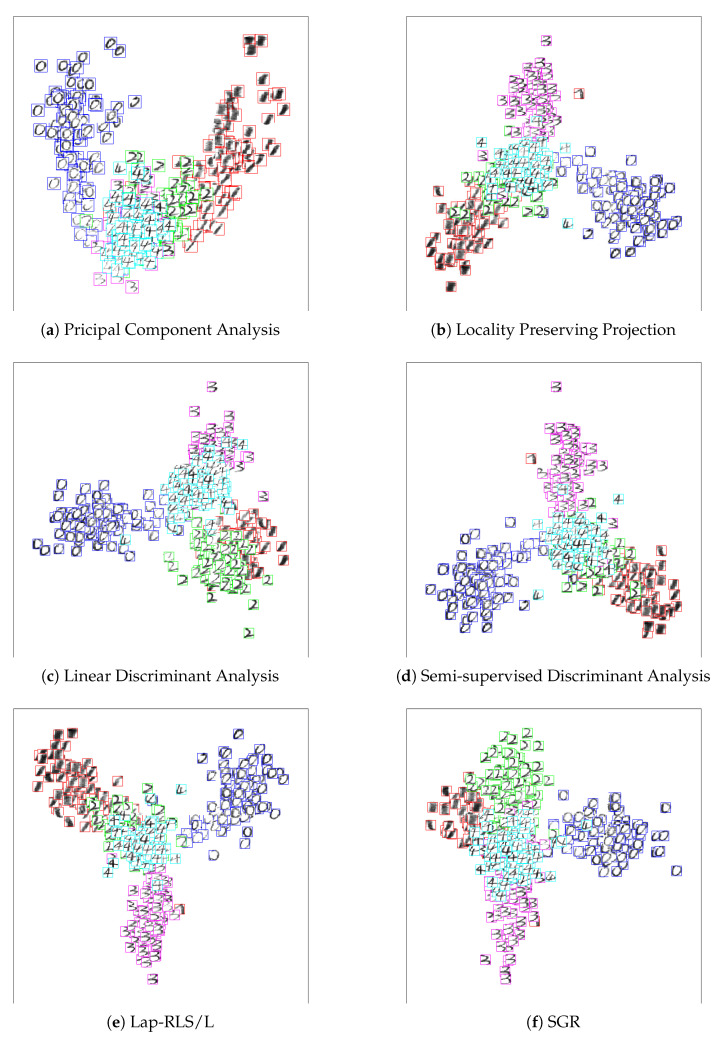
Visualization performance of different methods: five digits images from CASIA-HWDB: Principal Component Analysis (PCA), Locality Preserving Projection (LPP), Linear Discriminant Analysis (LDA), Semi-supervised Discriminant Analysis (SDA), Manifold Regularization (MR) and Sub-Graph Regularization (SGR).

In this subsection, we will demonstrate the visualization of the proposed method to show its superiority. In this study, we choose the digit and letter images of the first five classes from CASIA-HWDB dataset for experiment, where we randomly select 20 data and 80 data in each class to formulate a labeled set and an unlabeled set, respectively. The rest are used to formulate testing data. We then project the test set on the 2D subspace by utilizing a 2D projection matrix for visualization. Since the out-of-sample extension of the proposed SGR and MR are derived from the regression problem, we perform PCA operator on the projection data of $V^{T}X$ to reduce its dimensionality into two in order to handle the sub-manifold visualization problem. Then, the test data can be visualized on 2D subspace. The experiment results are shown in Figure 5 and Figure 6. From the experiment results, we can observe that SGR can obtain the better performance especially in CASIA-HWDB digit image data.

## 5. Conclusions

In this paper, we proposed a sub-graph-based SSL for image classification. The main contributions of the proposed work are as follows:

(1) We developed a doubly-stochastic *S* that measures the similarity between data points and anchors. The new updated *S* has probability means and can be viewed asa transition probability between data points and anchors. In addition, the new sub-graph is constructed by *S* in an efficient way and can preserve the geometry of data manifold. Simulation results verify the superiority of the proposed SGR;
(2) We also adopt a linear predictor for inferring the labels of new incoming data, which can handle out-of-sample problems. The computational complexity of this linear predictor is linear with the number of anchors; hence it is efficient. This shows that SGR can handle a large-scale dataset, which is quite practical;

From the above analysis, we can see that the main advantages for the proposed work is the effectiveness for handling the classification problems and that it needs less computational complexity for both graph construction and SSL. It can also handle out-of-sample problems based on a kernel regression on anchors. However, it also suffers the drawback that the parameters are not adaptive. In addition, the graph construction and SSL inference are in two different stages. Our future work can lie in developing a unified framework for optimization with adaptive adjusted parameters.

While the proposed work mainly focuses on image classification, our future work can also lie in handling other state-of-the-art applications, such as image retagging [38], and context classification in the natural language processing field [39,40]. 

## Figures and Tables

**Table 1:** The computational complexity of different stages. Semi-supervised learning (SSL).

The Proposed Method	The First Stage (Initialization)	The Second Stage (The Proposed Model)	The Third Stage (SSL)	Totals (Considering Large-Scale Data q≪l+u)
Computational Complexity	$O\left(q\left(l+u\right)\right)$	$O\left(q\left(l+u\right)\right)$	$O\left(q^{3}+q\left(l+u\right)\right)$	$O\left(q\left(l+u\right)\right)+O\left(q\left(l+u\right)\right)+$ $O\left(q^{3}+q\left(l+u\right)\right)\approx O\left(q\left(l+u\right)+q^{3}\right)$

**Table 2:** Information of different datasets.

Dataset	Database Type	Sample	Dim	Class	Train per Class	Test per Class
Extended Yale-B [30]	Face	16,123	1024	38	80%	20%
CMU-PIE [31]	Face	11,000	1024	68	80%	20%
COIL100 [32]	Object	7200	1024	100	58	14
ETH80 [33]	Object	3280	1024	80	33	8
USPS [34]	Hand-written digits	9298	256	10	800	remaining
CASIA-HWDB [35]	Hand-written letters	12,456	256	52	200	remaining

**Table 3:** Classification accuracies of the Yale-B dataset.

Methods	5% Training Labeled		10% Training Labeled		15% Training Labeled		20% Training Labeled	
	Unlabeled	Test	Unlabeled	Test	Unlabeled	Test	Unlabeled	Test
SVM	53.1 ± 1.1	52.7 ± 1.0	68.8 ± 2.0	67.7 ± 0.6	75.2 ± 1.1	73.7 ± 1.3	80.0 ± 1.8	78.8 ± 1.2
MR	59.0 ± 1.2	58.5 ± 1.3	70.3 ± 1.1	69.4 ± 0.5	76.4 ± 1.3	74.9 ± 1.5	80.7 ± 1.3	79.0 ± 1.1
LGC	64.7 ± 1.0		71.8 ± 1.1		76.4 ± 4.2		80.8 ± 1.0	
SLP	65.6 ± 2.3		73.9 ± 1.0		78.0 ± 1.8		81.8 ± 1.0	
LNP	64.9 ± 1.3	53.8 ± 2.7	72.0 ± 1.2	71.2 ± 0.4	78,0 ± 2.4	76.6 ± 2.1	81.6 ± 1.0	80.0 ± 1.4
AGR	66.6 ± 1.5	65.8 ± 1.3	74.3 ± 1.2	72.2 ± 0.4	78.1 ± 1.5	77.3 ± 1.7	83.0 ± 1.2	80.0 ± 4.5
EAGR	66.9 ± 0.8	66.5 ± 1.8	74.4 ± 1.1	73.2 ± 1.5	78.0 ± 1.5	77.2 ± 1.9	84.4 ± 2.4	83.6 ± 3.1
SGR	69.9 ± 0.4	67.2 ± 1.0	75.7 ± 1.1	74.0 ± 3.3	79.4 ± 1.0	78.3 ± 1.1	86.3 ± 2.5	82.8 ± 2.4

**Table 4:** Classification accuracies of the CMU-PIE dataset.

Methods	5% Training Labeled		10% Training Labeled		15% Training Labeled		20% Training Labeled	
	Unlabeled	Test	Unlabeled	Test	Unlabeled	Test	Unlabeled	Test
SVM	42.5 ± 1.3	41.5 ± 1.1	56.8 ± 2.2	55.8 ± 1.5	64.6 ± 1.2	63.8 ± 1.8	69.3 ± 1.7	68.9 ± 1.2
MR	47.8 ± 1.1	46.7 ± 1.6	59.3 ± 1.8	58.8 ± 1.3	65.6 ± 1.6	64.5 ± 1.6	69.9 ± 1.4	69.1 ± 1.4
LGC	53.5 ± 1.6		60.3 ± 1.7		66.5 ± 2.8		70.5 ± 1.3	
SLP	55.3 ± 1.9		63.4 ± 1.8		67.2 ± 1.9		70.9 ± 1.3	
LNP	55.2 ± 1.2	54.8 ± 1.9	62.9 ± 1.5	61.8 ± 0.9	68,3 ± 2.7	67.3 ± 2.3	71.1 ± 1.2	71.0 ± 1.6
AGR	56.4 ± 1.4	55.3 ± 1.8	64.8 ± 1.3	64.7 ± 0.5	68.5 ± 2.1	66.9 ± 1.8	72.8 ± 1.7	71.3 ± 3.5
EAGR	57.2 ± 1.0	56.4 ± 1.6	64.4 ± 1.2	63.7 ± 1.9	68.4 ± 1.8	67.7 ± 2.3	73.1 ± 2.0	72.4 ± 2.7
SGR	59.0 ± 0.7	58.4 ± 1.3	65.6 ± 1.2	64.6 ± 1.9	69.8 ± 1.6	67.9 ± 1.6	75.0 ± 2.4	73.9 ± 2.3

**Table 5:** Classification accuracies of the COIL100 dataset.

Methods	5% Training Labeled		10% Training Labeled		15% Training Labeled		20% Training Labeled	
	Unlabeled	Test	Unlabeled	Test	Unlabeled	Test	Unlabeled	Test
SVM	83.6 ± 0.9	83.2 ± 0.8	88.5 ± 0.8	86.6 ± 0.8	91.8 ± 0.8	91.4 ± 0.7	95.3 ± 0.8	94.5 ± 1.6
MR	83.7 ± 1.0	83.4 ± 0.9	89.0 ± 0.9	87.3 ± 0.9	92.1 ± 0.8	91.6 ± 0.9	95.3 ± 0.7	94.7 ± 1.3
LGC	85.5 ± 0.8		89.3 ± 0.9		92.4 ± 0.8		95.5 ± 0.6	
SLP	86.4 ± 0.7		89.3 ± 0.9		92.8 ± 0.6		95.6 ± 0.8	
LNP	86.5 ± 0.7	85.6 ± 0.7	89.6 ± 0.9	88.7 ± 0.7	92.9 ± 0.7	92.4 ± 0.8	95.8 ± 0.7	95.1 ± 1.3
AGR	86.5 ± 0.6	85.8 ± 0.9	90.9 ± 0.9	88.8 ± 0.8	93.3 ± 0.6	92.7 ± 0.9	95.8 ± 0.7	95.3 ± 1.4
EAGR	86.6 ± 0.7	85.7 ± 1.3	89.9 ± 0.9	89.0 ± 1.5	93.2 ± 0.6	92.7 ± 1.5	96.0 ± 0.7	95.2 ± 0.9
SGR	87.0 ± 0.6	86.7 ± 1.0	91.8 ± 0.9	89.7 ± 0.8	94.7 ± 0.6	93.2 ± 0.8	97.0 ± 0.6	95.6 ± 0.9

**Table 6:** Classification accuracies of the ETH80 dataset.

Methods	5% Training Labeled		10% Training Labeled		15% Training Labeled		20% Training Labeled	
	Unlabeled	Test	Unlabeled	Test	Unlabeled	Test	Unlabeled	Test
SVM	61.1 ± 1.3	59.4 ± 0.3	71.1 ± 1.9	70.2 ± 2.0	75.9 ± 1.5	75.3 ± 3.1	78.9 ± 2.0	77.9 ± 2.5
MR	62.3 ± 0.8	60.0 ± 0.2	71.7 ± 2.0	71.0 ± 2.7	76.2 ± 1.0	75.3 ± 2.8	78.9 ± 1.9	78.3 ± 2.5
LGC	65.7 ± 1.4		73.5 ± 1.4		76.8 ± 1.5		79.0 ± 1.7	
SLP	65.9 ± 1.5		73.9 ± 1.2		76.9 ± 1.6		79.3 ± 1.8	
LNP	64.9 ± 0.9	62.2 ± 0.2	73.4 ± 2.0	71.4 ± 2.6	76.7 ± 1.1	76.0 ± 2.6	79.0 ± 1.8	78.5 ± 2.0
AGR	66.4 ± 1.6	65.1 ± 0.2	75.0 ± 1.7	72.2 ± 2.2	76.9 ± 1.7	76.1 ± 2.5	79.6 ± 2.0	78.9 ± 1.9
EAGR	68.2 ± 1.7	67.7 ± 2.1	74.9 ± 1.4	74.2 ± 1.9	77.3 ± 1.7	77.0 ± 1.9	80.0 ± 2.2	79.4 ± 2.8
SGR	69.4 ± 1.9	67.2 ± 0.1	74.0 ± 1.3	74.2 ± 2.2	77.5 ± 1.9	77.3 ± 1.8	79.8 ± 22	79.0 ± 2.2

**Table 7:** Classification accuracies of the ETH80 dataset.

Methods	5% Training Labeled		10% Training Labeled		15% Training Labeled		20% Training Labeled	
	Unlabeled	Test	Unlabeled	Test	Unlabeled	Test	Unlabeled	Test
SVM	71.7 ± 0.7	70.6 ± 1.5	77.9 ± 0.7	77.8 ± 0.2	91.9 ± 4.4	90.9 ± 4.2	96.1 ± 1.9	95.7 ± 0.9
MR	74.1 ± 0.7	73.0 ± 1.5	80.9 ± 0.8	79.8 ± 0.1	92.6 ± 3.4	91.7 ± 3.4	96.1 ± 2.2	95.0 ± 1.0
LGC	74.7 ± 0.7		87.1 ± 0.8		94.6 ± 3.3		96.5 ± 2.3	
SLP	75.0 ± 0.5		89.7 ± 0.7		95.4 ± 3.0		96.5 ± 2.3	
LNP	76.5 ± 0.6	74.8 ± 0.8	92.0 ± 0.7	90.8 ± 0.5	95.5 ± 3.4	95.0 ± 3.4	96.9 ± 2.5	96.5 ± 0.9
AGR	78.7 ± 0.6	76.1 ± 0.7	93.6 ± 0.7	92.6 ± 0.7	96.0 ± 2.4	95.8 ± 2.4	97.1 ± 2.8	96.7 ± 0.9
EAGR	79.9 ± 0.6	79.4 ± 1.2	93.6 ± 0.7	92.9 ± 1.1	96.3 ± 3.6	95.5 ± 3.5	97.2 ± 1.7	96.3 ± 2.2
SGR	80.7 ± 0.5	79.7 ± 0.7	95.0 ± 0.5	93.3 ± 0.8	97.2 ± 3.1	96.2 ± 3.1	97.4 ± 1.5	97.3 ± 0.7

**Table 8:** Classification accuracies of the CASIA-HWDB dataset.

Methods	5% Training Labeled		10% Training Labeled		15% Training Labeled		20% Training Labeled	
	Unlabeled	Test	Unlabeled	Test	Unlabeled	Test	Unlabeled	Test
SVM	56.8 ± 5.4	55.8 ± 0.6	65.7 ± 0.6	64.0 ± 1.7	79.0 ± 0.5	78.2 ± 4.0	83.4 ± 1.8	82.1 ± 1.9
MR	58.7 ± 3.3	57.3 ± 0.5	73.0 ± 0.6	62.0 ± 1.4	79.4 ± 0.6	78.4 ± 2.7	86.6 ± 1.9	85.5 ± 1.5
LGC	63.1 ± 2.4		76.1 ± 0.4		80.7 ± 0.5		88.1 ± 1.4	
SLP	63.4 ± 1.6		77.4 ± 0.4		85.3 ± 0.5		88.6 ± 1.7	
LNP	66.5 ± 1.4	64.8 ± 0.6	78.5 ± 0.5	77.5 ± 0.7	85.9 ± 0.5	84.8 ± 1.7	89.2 ± 1.7	90.6 ± 8.2
AGR	72.0 ± 0.9	71.0 ± 0.6	80.9 ± 2.8	77.8 ± 0.6	87.2 ± 0.5	86.4 ± 1.6	91.8 ± 1.6	90.0 ± 4.1
EAGR	74.9 ± 0.7	74.4 ± 1.2	78.6 ± 3.3	78.0 ± 3.1	87.6 ± 0.4	87.2 ± 1.0	91.6 ± 1.8	91.2 ± 2.2
SGR	75.3 ± 0.7	73.6 ± 0.5	83.6 ± 2.2	80.3 ± 0.6	88.7 ± 0.3	86.5 ± 1.6	93.2 ± 1.7	91.7 ± 3.3
